# Cholesterol loading suppresses the atheroinflammatory gene polarization of human macrophages induced by colony stimulating factors

**DOI:** 10.1038/s41598-021-84249-y

**Published:** 2021-03-01

**Authors:** Jani Lappalainen, Nicolas Yeung, Su D. Nguyen, Matti Jauhiainen, Petri T. Kovanen, Miriam Lee-Rueckert

**Affiliations:** 1 Wihuri Research Institute, Helsinki, Finland; 2 Minerva Foundation Institute for Medical Research, Biomedicum, Helsinki, Finland

**Keywords:** Biochemistry, Cell biology, Diseases, Atherosclerosis

## Abstract

In atherosclerotic lesions, blood-derived monocytes differentiate into distinct macrophage subpopulations, and further into cholesterol-filled foam cells under a complex milieu of cytokines, which also contains macrophage-colony stimulating factor (M-CSF) and granulocyte–macrophage-colony stimulating factor (GM-CSF). Here we generated human macrophages in the presence of either M-CSF or GM-CSF to obtain M-MØ and GM-MØ, respectively. The macrophages were converted into cholesterol-loaded foam cells by incubating them with acetyl-LDL, and their atheroinflammatory gene expression profiles were then assessed. Compared with GM-MØ, the M-MØ expressed higher levels of *CD36*, *SRA1*, and *ACAT1*, and also exhibited a greater ability to take up acetyl-LDL, esterify cholesterol, and become converted to foam cells. M-MØ foam cells expressed higher levels of *ABCA1* and *ABCG1*, and, correspondingly, exhibited higher rates of cholesterol efflux to apoA-I and HDL_2_. Cholesterol loading of M-MØ strongly suppressed the high baseline expression of *CCL2*, whereas in GM-MØ the low baseline expression *CCL2* remained unchanged during cholesterol loading. The expression of *TNFA*, *IL1B*, and *CXCL8* were reduced in LPS-activated macrophage foam cells of either subtype. In summary, cholesterol loading converged the CSF-dependent expression of key genes related to intracellular cholesterol balance and inflammation. These findings suggest that transformation of CSF-polarized macrophages into foam cells may reduce their atheroinflammatory potential in atherogenesis.

## Introduction

Macrophages substantially impact the development and progression of atherosclerosis through their diverse roles in cholesterol metabolism and by regulating innate immune responses^1–3^. Formation of an atherosclerotic lesion is characterized by the accumulation of LDL-derived as well as lipoprotein remnant-derived cholesterol in the lesional macrophages leading to foam cell formation and chronic inflammatory responses in the arterial intima^4–6^.

Stimulation of cholesterol efflux from macrophage foam cells by HDL can significantly compensate for the influx of cholesterol, and such cholesterol efflux capacity of HDL is considered the main cardioprotective function of this class of lipoproteins^7^. Cholesterol efflux from macrophage foam cells occurs via several pathways, quantitatively the most effective mechanism involves the ATP-binding cassette (ABC) transporters A1 and G1, which upon interaction with lipid-poor and mature HDL, respectively, trigger a cascade of events associated with the release of cholesterol and phospholipids from the foam cells^8,9^. By disrupting the specialized cholesterol and sphingomyelin-rich lipid rafts, which serve as platforms in the macrophage plasma membrane for inflammatory signaling pathways, cholesterol efflux is associated with anti-inflammatory effects and proper immune responses^10^. Such link between cholesterol efflux and inflammation is supported by data showing that cholesterol accumulation in the plasma membrane of Abca1^−/−^, Abcg1^−/−^, and Abca1^−/−^Abcg1^−/−^ macrophages increases signaling of Toll-like receptors (TLR), thereby enhancing the inflammatory response to LPS and other TLR ligands^11–13^. Furthermore, it was reported that ABCA1 can also function as an anti-inflammatory signaling receptor in macrophages through activation of STAT3, an effect independent of cholesterol efflux induction^14^.

Macrophage colony-stimulating factor (M-CSF) and granulocyte–macrophage colony-stimulating factor (GM-CSF) are soluble glycoproteins widely expressed in the arterial intima^15^. They regulate the differentiation of mononuclear phagocytic cells into mature macrophages and also affect their functional properties^16^. During atherogenesis, exposure to CSFs and other polarizing factors modulates the generation of macrophage populations with divergent genomic signatures and functional properties that may impact their atherogenic roles^15^. Thus, upon exposure of monocytes to M-CSF or GM-CSF, 2 major macrophage subtypes are generated which exhibit distinct cell morphologies, antigen expression, and functional responses, although also several overlapping features exist^17–19^.

Even though various macrophage subtypes exhibit functional diversity in vascular biology, they have been only partially mapped^20^. Importantly, the pre-foam-cell macrophages resemble strong phenotypic plasticity when activated, with a spectrum ranging from pro-inflammatory to wound-healing and regression-driving phenotypes^1^. As such, any dichotomous classification of macrophages only represents the opposite ends of a wide spectrum of macrophage phenotypes, yet it provides a useful concept to discuss the role of macrophages in inflammation and its resolution in atherosclerotic plaques^21,22^. Moreover, given the complexity of the tissue microenvironment and the ability of macrophages to undergo metabolic adaptations to sustain their activities, a static vision of macrophage polarization generated in vitro may not fully reflect the dynamic and tissue-specific macrophage polarization in vivo^23^. Interestingly, however, a macrophage subpopulation predominating in human atherosclerotic lesions has been shown to resemble macrophages generated in vitro in the presence of M-CSF and IL-10^19^.

The transformation of macrophages into foam cells plays a central role in atherogenesis. This process also strongly affects their gene expression profile. Therefore, investigation of the mechanism by which human monocyte-derived macrophages generated via different CSF treatments in vitro react to cholesterol loading becomes of particular interest to understand better the potential mechanisms operating in vivo. In mice, genes linked to lipid metabolism, complement activation, or lysosomal function are differentially overexpressed in cholesterol-loaded macrophages indicating that the cholesterol cargo is capable of regulating functional macrophage properties implicated in atherogenesis^24^. However, functional correlates of mouse and human macrophages and their representative cell subtypes have not been fully defined, so presenting a barrier for a comprehensive understanding of macrophage subpopulations in human atherogenesis.

Since CSF-macrophage subpopulations have already been identified in human coronary arteries^19^ and various subtypes are being involved in distinct roles regarding progression or regression of atherosclerotic plaques^25^, it is pertinent to pose a question of whether the cholesterol-rich environment, such as prevailing in the atherosclerotic intima, can modulate the CSF-induced gene polarization of human macrophages. To address this, we investigated in vitro the effect of cholesterol loading on the expression of selected genes which play essential roles in the regulation of cholesterol balance and inflammation in human macrophage foam cells differentiated solely under the influence of either M-CSF or GM-CSF. Our results demonstrate that the transformation of human monocyte-derived macrophages into foam cells converges the gene expression profiles of the macrophage subtypes polarized with either CSF, thereby reducing their CSF-dependent atheroinflammatory polarization.

## Results

### Phenotypic macrophage markers are expressed at different levels in the presence of M-CSF or GM-CSF

To gain knowledge about the effect of CSFs on human macrophage gene expression profile, we analyzed the cells (i) after monocyte differentiation into macrophages with either M-CSF or GM-CSF (“M-MØ and GM-MØ”), (ii) after cholesterol loading of the generated macrophages with acetyl-LDL to yield “Foam cells M-MØ and GM-MØ”, and (iii) after cholesterol efflux of the generated foam cell macrophages with cholesterol acceptors to yield “Regressing foam cells M-MØ and GM-MØ” (Supplementary Fig. S1).

After 6 days of monocyte differentiation in the presence of either CSF, the cells were immunostained for the macrophage markers CD68 and CD14. As previously reported in human monocyte-derived macrophages^17,19,26^, CD14^+^ cells predominated in cultures incubated with M-CSF, while CD68 was uniformly expressed in either CSF culture, indicating a phenotypic conversion into 2 major macrophage subsets (Supplementary Fig. S2). Because GM-CSF-treated monocytes are widely used as a model for the development of dendritic cells^16^, we immunophenotyped the dendritic cell markers CD11c and CCR7 in cells differentiated with GM-CSF in the absence or presence of IL-4, the latter capable of inducing dendritic cell formation *in vitro*^27^. In line with the immunostaining data, monocytes incubated with GM-CSF were differentiated into CD68^+^ cells which also expressed the CD14 antigen (Supplementary Fig. S3, top panel left); however, when monocytes were differentiated with GM-CSF and IL-4, the generated cells were CD68^+^/CD14^−^ (Supplementary Fig. S3, top panel right). These cells also expressed the dendritic cell marker CD11c and some expressed CCR7, whereas the cells differentiated in the absence of IL-4 were CD11c^+^/CCR7^−^ (Supplementary Fig. S3, top panel left). Besides, overlaid histograms were generated to compare antigen expression intensity for CD14, CD11c, and CCR7 (Supplementary Fig. S3, bottom panel). These data confirm previous observations indicating that the expression patterns of cell surface markers in human macrophages and dendritic cells partially overlap^28^. Thus, in the presence of IL-4, the GM-CSF generated cells with characteristics of immature dendritic cells, as shown by negative staining for CD14, and only a few mature dendritic cells expressing the CCR7 antigen. Overall, the data indicated that the present experimental culture setting generated 2 subtypes of cells identified as macrophages rather than dendritic cells. Therefore, human monocytes differentiated either with M-CSF or GM-CSF were designated as the macrophage subtypes M-MØ and GM-MØ, respectively.

### Gene expression profile characterizes M-MØ as more proatherogenic and pro-inflammatory than GM- MØ

**Figure 1:**
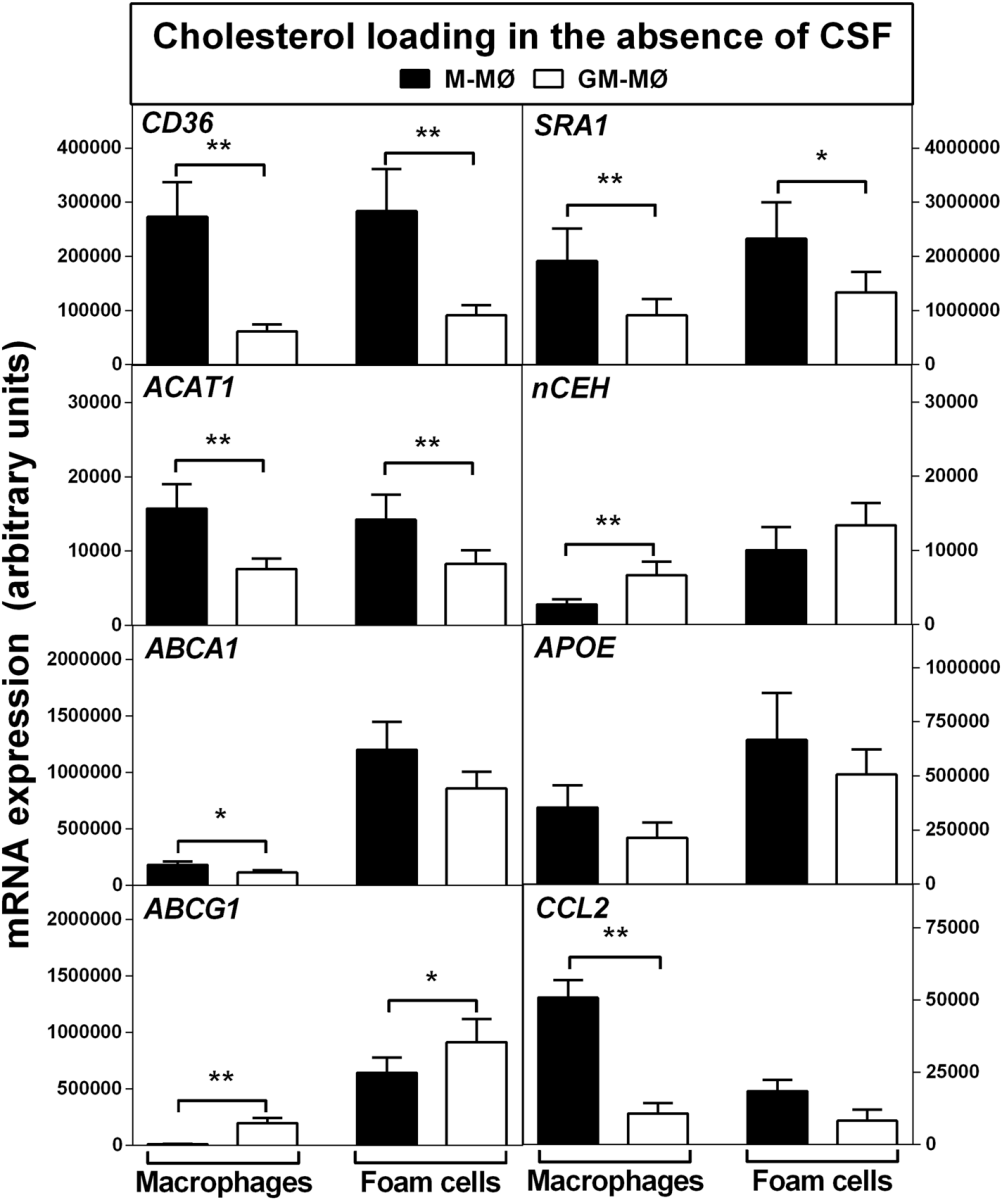
Expression of genes responsible for cholesterol accumulation and inflammation in M-CSF or GM-CSF-polarized macrophages before and after their conversion into foam cells in the absence of CSF. Gene expression levels were determined by quantitative RT-PCR in M-MØ and GM-MØ before (Macrophages) and after (Foam cells) cholesterol loading with acetyl-LDL (25 µg/mL). Data are presented as means ± SEM of 10 individual blood donors and triplicate incubations for each studied gene. *p < 0.05 and **p < 0.01 denote statistically significant difference between the expression levels in M-MØ and GM-MØ, both before and after cholesterol loading, by Wilcoxon Signed-Rank test for paired samples.

To characterize further the phenotypes of macrophages polarized in the sole presence of either CSF, expression of relevant genes related to cholesterol uptake, cholesterol efflux, and inflammation was examined. Accordingly, we evaluated the mRNA expression of 8 key molecules controlling lipid uptake (CD36, SRA1), HDL-facilitated cholesterol efflux (ABCA1, ABCG1, apoE) and those affecting intracellular cholesterol traffic pathways (ACAT1, nCEH), as well as CCL2, a known amplifier of the inflammatory response. The basal gene profiles of the freshly differentiated cells indicated stronger expression in the M-MØ for the majority (5 out of 8) of the studied genes (Fig. 1, see “Macrophages” columns) reflecting greater plasticity of macrophages which had been polarized in M-CSF medium. In these cells, higher expression of genes encoding scavenger receptors for modified-LDL uptake (*CD36* and *SRA1*) and the cholesterol esterification enzyme (*ACAT1*), and lower expression of the gene related to cholesteryl ester hydrolysis (*nCEH*) suggested that the M-MØ macrophage subtype was particularly prone to foam cell formation. Analysis of the expression of genes related to cholesterol efflux indicated that levels of *ABCA1* and *APOE* (encoding apolipoprotein E, which induces ABCA1-mediated cholesterol efflux^29^) were higher while that of *ABCG1* was markedly lower (~ 20-fold) in M-MØ, as compared to GM-MØ. In agreement with a recent report^30^, M-CSF polarization also induced roughly fivefold higher expression of *CCL2*, a macrophage inflammatory marker critical for monocyte recruitment. Such contrasting CSF-dependent regulatory effects on *ABCG1* and *CCL2* have also been reported in monocytes differentiated with M-CSF or GM-CSF in the presence of IL-10 and serum, i.e. in a medium containing a myriad of macrophage-polarizing factors^19^. Considering the reported anti-inflammatory role of ABCG1 in macrophages^31^ and the well-known effect of CCL2 as a chemoattractant for amplifying inflammatory response^32^, monocytes differentiated under the sole influence of M-CSF had gained pro-inflammatory traits. Of note, M-CSF or GM-CSF was the only essential growth factor present in the minimal serum-free culture medium employed and, evidently, sufficient for the observed cellular effects.

### Cholesterol loading converges gene expression in CSF-polarized macrophages

Next, we investigated how the divergent gene profile of the CSF-differentiated macrophage subtypes is modified upon macrophage conversion into foam cells for the fact that the macrophage subtypes appeared to have distinct foam cell-forming and pro-inflammatory potentials. For this purpose, the macrophages were incubated with acetyl-LDL in the absence of CSFs to avoid any reported effects of respective CSF on the expression of scavenger receptors^33,34^, and the gene profiles of the foam cells were compared with those assessed in the basal macrophages.

As shown in Fig. 1 (see “Foam cells” columns), cholesterol loading did not change the mRNA expression levels of *CD36*, *SRA1*, or *ACAT1* in either macrophage subtype, while induction of *nCEH* in both subtypes of foam cells reduced the difference observed in *nCEH* expression between M-MØ and GM-MØ at the basal stage (top panels, compare “Macrophages” vs “Foam cells” columns). Interestingly, cholesterol loading increased the expression of 3 genes related to cholesterol efflux (*ABCA1*, *ABCG1*, *APOE*) and reduced the expression of *CCL2*, a gene whose high expression level signifies inflammation^32^. Accordingly, all observed changes in the gene expression levels dampened the divergences between the basal M-MØ and GM-MØ (bottom panels, compare “Macrophages” vs “Foam cells” columns), so resulting in a more convergent gene profile in the 2 foam cell subtypes. Moreover, the pro-inflammatory features observed in M-MØ at the basal stage were strongly suppressed in response to cholesterol loading as indicated by the 60-fold upregulation of *ABCG1* and twofold downregulation of *CCL2*. In contrast, immunophenotyping of surface antigens demonstrated that the respective CSF-specific markers CD68^+^/CD14^+^/CD11c^+^ of the 2 macrophage subtypes remained unaltered after cholesterol loading strongly indicating that the foam cells maintained their respective CSF phenotypic signature (Supplementary Fig. S4).

**Figure 2:**
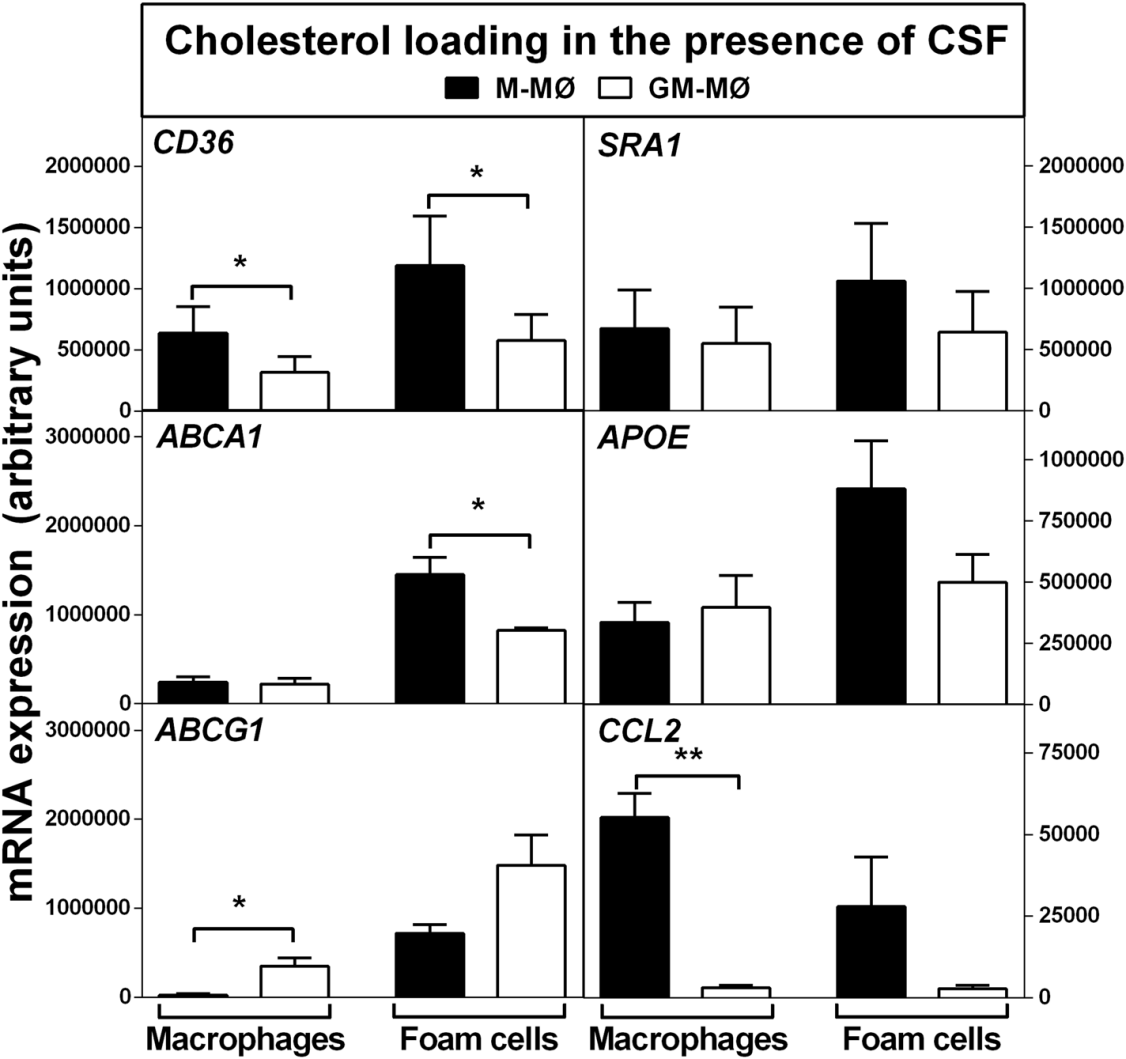
Expression of genes responsible for cholesterol accumulation and inflammation in M-CSF or GM-CSF-polarized macrophages before and after their conversion into foam cells in the presence of CSF. Gene expression levels were determined by quantitative RT-PCR in M-MØ and GM-MØ before (Macrophages) and after (Foam cells) cholesterol loading with acetyl-LDL (25 µg/mL) in the presence of the respective CSF. Data are presented as means ± SEM of 5 individual blood donors and triplicate incubations for each studied gene. *p < 0.05 and **p < 0.01 denote statistically significant differences between the expression levels in M-MØ and GM-MØ, both before and after cholesterol loading, by Wilcoxon Signed-Rank test for paired samples.

In a separate control experiment, we examined whether the observed changes induced by cholesterol loading in M-MØ and GM-MØ phenotypes also occurred when either CSF was included in the culture media during incubation in the presence of acetyl-LDL. As shown in Fig. 2, changes induced in the gene expression of the foam cell subtypes were similar to those observed in cells incubated in the absence of CSF (see Fig. 1). Importantly, the strong shifts in the expression of *ABCG1* (~ 30-fold increase) and *CCL2* (~ twofold decrease) were observed in M-MØ foam cells, again, in the presence of CSF. Overall, these data provide additional support to the notion that, while both CSFs differentially target key regulatory genes involved in cholesterol efflux and inflammation during macrophage polarization, the intracellular cholesterol accumulation, but not the type of CSF, modulates the expression of these genes during the conversion of the CSF macrophage subtypes into foam cells. Thus, we considered justified that after the CSF-driven period of macrophage differentiation, subsequent cell incubations were performed in media devoid of CSF.

**Figure 3:**
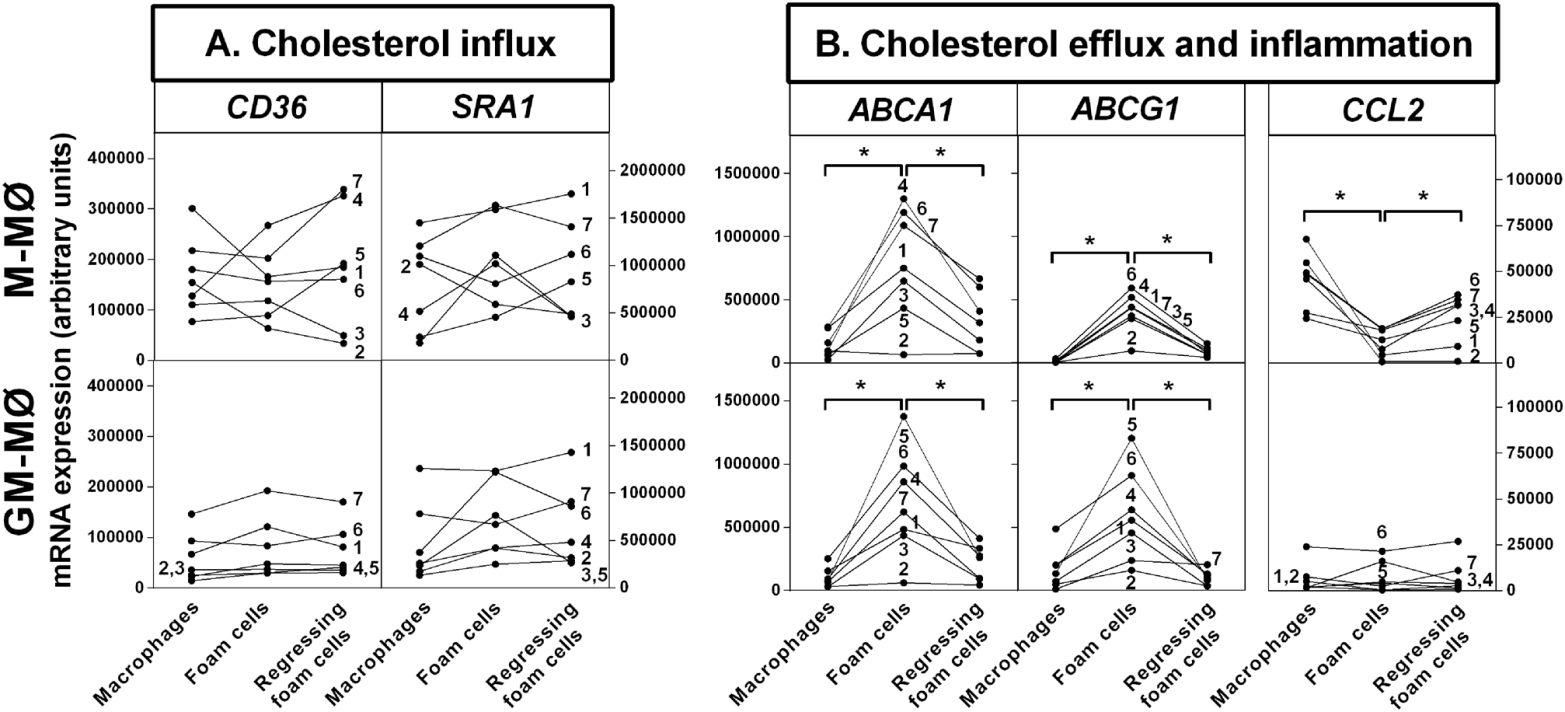
Gene expression of scavenger receptors, ABC transporters, and CCL2 in M-MØ and GM-MØ after cholesterol influx and efflux. M-MØ and GM-MØ were analyzed for expression of (**A**) CD36 and SRA1, and (**B**) ABCA1, ABCG1, and CCL2 before (Macrophages) and after (Foam cells) incubation with acetyl-LDL, and after cholesterol efflux to an unfractionated pool of human plasma (2.5%) (Regressing foam cells), as described in Supplementary Fig. S1. Data from 7 individual blood donors are presented as means of triplicate incubations for the mRNA values at each data point. *p < 0.05 by Wilcoxon Signed-Rank test for paired samples.

We also examined the expression of genes related to cholesterol balance and inflammation in cholesterol-loaded M-MØ and GM-MØ after the foam cells were incubated in the presence of human normolipidemic plasma (“Regressing foam cells”). This analysis was performed using CSF-polarized macrophages derived from monocytes isolated from the blood of 7 healthy donors (Fig. 3). Neither cholesterol loading nor cholesterol efflux modified *CD36* or *SRA1* expression in either macrophage subtype and, accordingly, higher levels of these scavenger receptors remained in M-MØ after each incubation step (Fig. 3A). In contrast, consistent with the sterol-sensitive regulation of ABC transporters^35^, cholesterol loading increased and cholesterol efflux decreased *ABCA1* and *ABCG1* expression in either MØ subtype (Fig. 3B). Although the CSF-specific gene responses were highly variable between donors, as a rule, macrophages expressing more *ABCA1* also expressed more *ABCG1*, irrespective of the CSF subtype. Thus, in Donor #5 upregulation of ABC genes in response to cholesterol loading was much lower when macrophages were polarized with M-CSF than with GM-CSF, whereas in Donor #2, #4, and #6 similar responses were observed to either CSF (low in Donor #2; high in Donors #4 and #6). Notably, concerning the inflammatory marker CCL2, its expression in M-MØ foam cells was significantly decreased by cholesterol loading, and cholesterol removal easily reversed this effect (Fig. 3B). In contrast, the weak basal expression of *CCL2* in GM-MØ cells remained unaffected by cholesterol loading and unloading. These data indicated that *CCL2* expression was particularly responsive to cellular cholesterol homeostasis in macrophages polarized with M-CSF. Since circulating monocytes consist of a heterogeneous pool of at least 3 distinct subsets^36^, the observed donor-dependent variation in the expression of ABC genes may be attributed to the phenotypic state of the monocytes prior polarization reflecting a variable abundance of CSF receptors.

### The gene expression response to LPS of inflammatory cytokines is reduced in cholesterol-loaded macrophages

**Figure 4:**
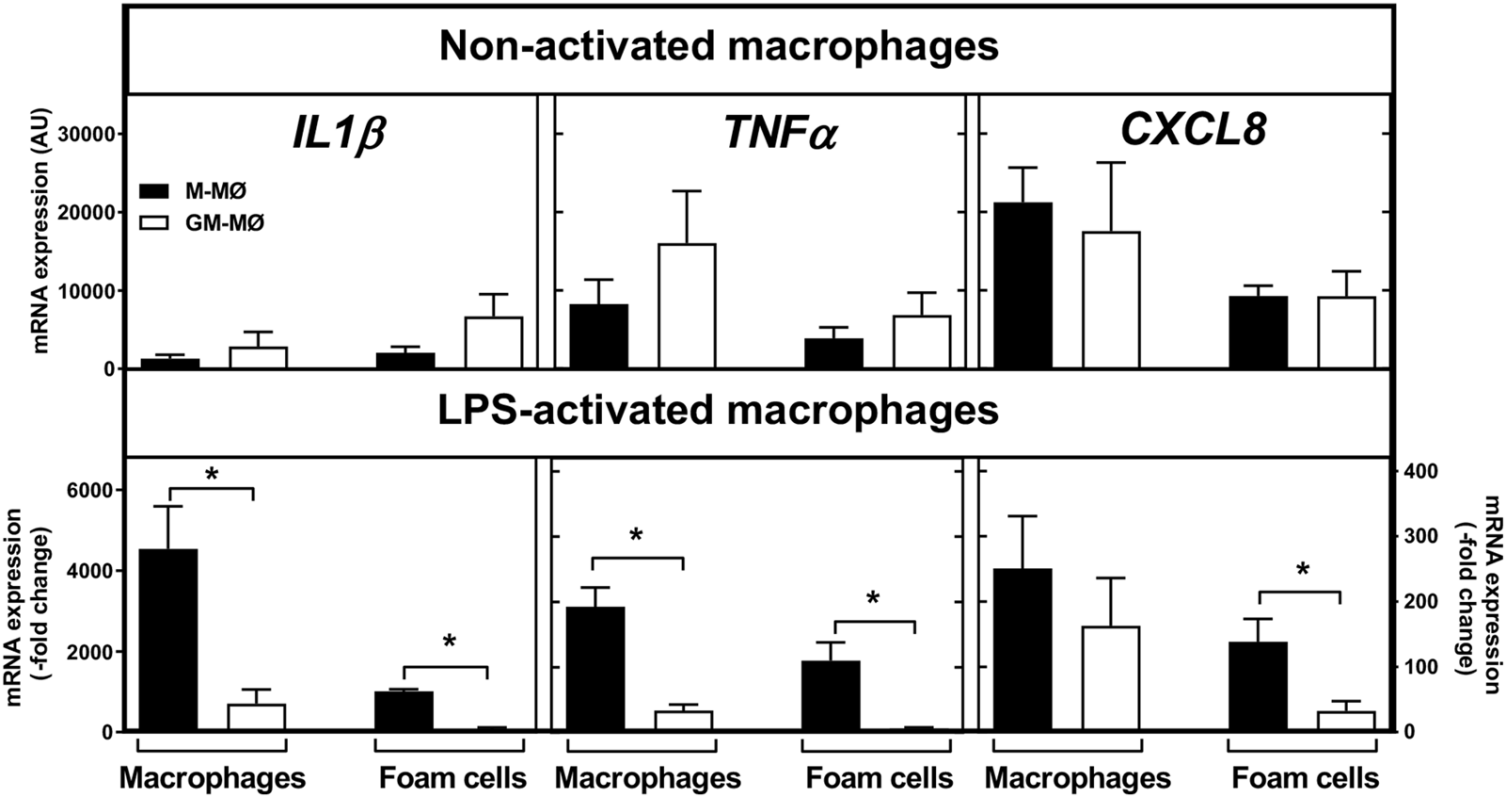
Gene expression of inflammatory cytokines in cholesterol-loaded M-MØ and GM-MØ after LPS activation. The mRNA expression of 3 key cytokines with proinflammatory properties (IL-1β, TNF-α, and CXCL8) by activated human macrophages was evaluated after incubation of non-loaded macrophages (“Macrophages”) and cholesterol-loaded macrophages (“Foam Cells”) of each CSF subtype for 3 h with LPS. The cytokine expression in LPS-activated macrophages (bottom panels) was expressed as fold-changes relative to their levels in the respective non-activated MØ subtypes (top panels). For each condition, data are presented as means ± SEM of 3 individual blood donors, and triplicate incubations for each condition. *p < 0.05 denotes statistically significant differences determined by Wilcoxon Signed-Rank test for paired samples.

LPS-induced activation of CSF-polarized murine and human macrophages has been reported to result in divergent cytokine secretion profiles^37,38^. Thus, we investigated further whether the regulatory effect of foam cell formation on *CCL2* expression observed in M-CSF-polarized macrophages also applies to other selected relevant inflammatory cytokines. For this aim, mRNA expression of *IL1B*, *TNFA*, and *CXCL8*, which are significantly expressed by LPS-activated human macrophages^39^, was assessed in non-loaded (“Macrophages”) and cholesterol-loaded (“Foam Cells”) of either CSF subtype, which had been incubated for 3 h in the absence or presence of LPS (Fig. 4).

In the absence of LPS (non-activated cells), no significant difference in the basal expression of the selected cytokines was found between the 2 non-loaded macrophage subtypes (Fig. 4, top panel) and, moreover, cholesterol loading induced similar expression trends in either foam cell subtype by slightly increasing (*IL1B*) or decreasing (*TNFA* and *CXCL8*) mRNA expression. Thus, overall, foam cell formation appeared to suppress the expression of inflammatory cytokines in both CSF macrophage subtypes. We, next, examined cytokine expression in the non-loaded and cholesterol-loaded M-MØ and GM-MØ after incubation in the presence of LPS (activated cells), and data were expressed as fold-changes relative to those found in the respective type of non-activated cells (Fig. 4, bottom panel). We found that LPS induced the expression of *IL1B*, *TNFA*, and *CXCL8* in each CSF macrophage subtype irrespective of their cholesterol cargo, i.e. both in non-loaded and foam cells. Yet, foam cell conversion tended, again, to ameliorate cytokine expression in the LPS-activated macrophages (compare “Macrophages” and “Foam Cells” groups). These results indicate that cholesterol loading exerted a regulatory effect on macrophage inflammatory profile with a tendency to suppress the expression of, at least, the 3 selected cytokines. Given the well-known roles of these cytokines in acute inflammation, such an unpredicted result indicates that the worsening of inflammatory markers manifested in LPS-treated human macrophages was attenuated in the foam cell phenotype. Of interest, the cytokine response to LPS in the M-MØ subtype was markedly stronger and therefore, the expression levels of *IL1B*, *TNFA*, and *CXCL8* remained significantly higher in M-MØ foam cells despite their attenuation by cholesterol-loading (see bottom panel).

### Transcription factors show differential gene expression in cholesterol-loaded M-MØ and GM-MØ

**Figure 5:**
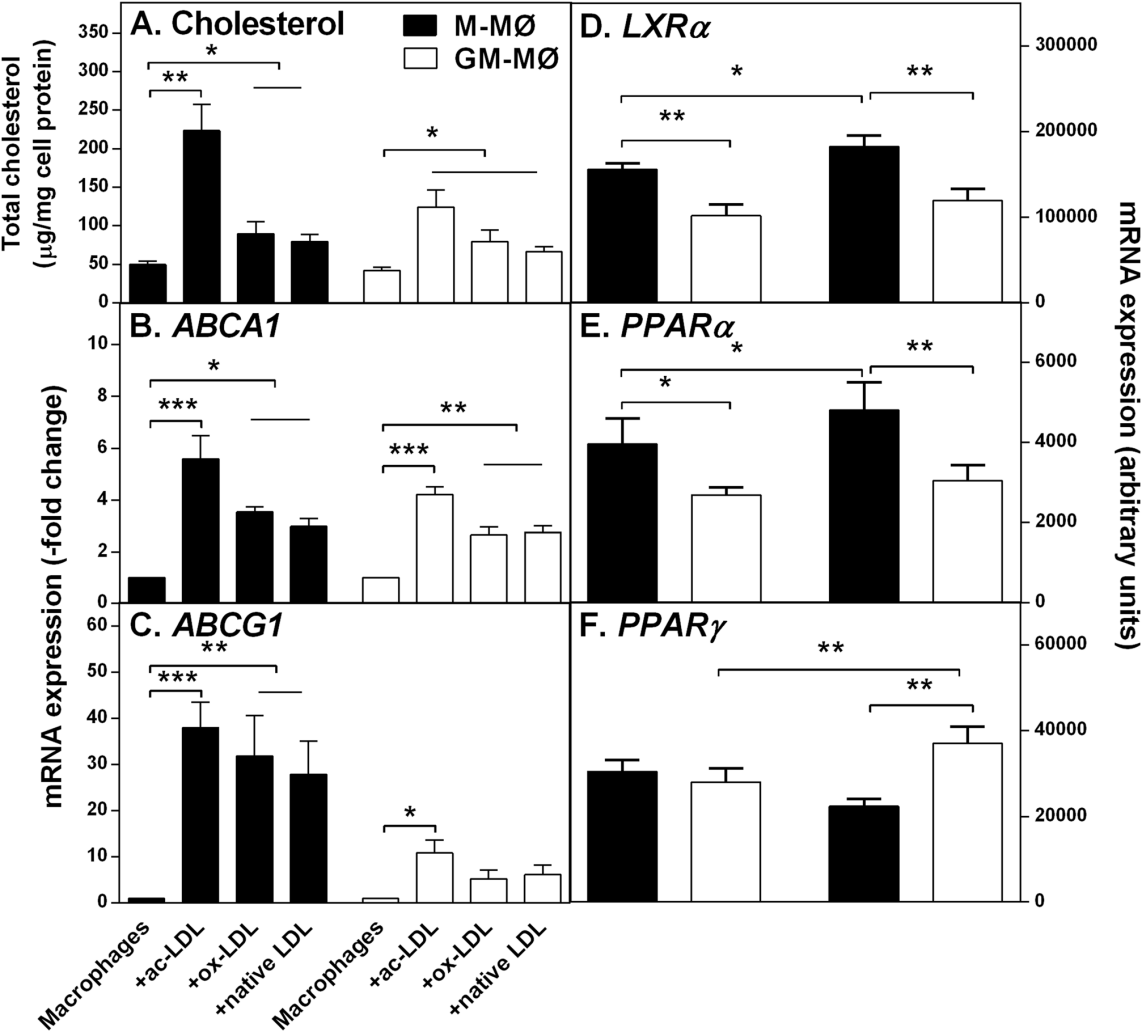
Effect of cholesterol loading on the gene expression of ABC transporters and related transcription factors in M-MØ and GM-MØ foam cells. M-MØ and GM-MØ were cholesterol-loaded for 24 h with 25 µg/mL of acetyl-LDL (ac-LDL), oxidized-LDL (ox-LDL), or native LDL. (**A**) Cholesterol was quantified by HPTLC, and expression of (**B**) *ABCA1* and (**C**) *ABCG1* was determined by quantitative RT-PCR. Data in panels (**B**) and (**C**) indicate mRNA expression levels relative to those in the non-loaded cells (Macrophages) (set at 1). Gene expression of (**D**) *LXRA*, (**E**) *PPARA*, and (**F**) *PPARG* was determined in acetyl-LDL generated foam cells of each subtype. Data in panels (**D**) to (**F**) indicate mRNA values expressed as arbitrary units. *p < 0.05, **p < 0.01, and ***p < 0.001 by one-way ANOVA. Data are presented as means ± SEM of 4–9 individual blood donors and triplicate incubations for each loading condition and gene of interest.

LXRs are known as cholesterol sensors that reciprocally regulate genes involved in cholesterol efflux and inflammation^40^. Importantly, LXRα and PPARα increase both *ABCA1* and *ABCG1* expression in macrophages^35^, while a recent report indicated that PPARγ induces *ABCG1* but decreases *ABCA1* expression^41^. To learn about mechanisms by which cholesterol cargo regulates the expression of ABC transporters in the 2 macrophage subtypes, we analyzed the cholesterol contents and mRNA levels of *ABCA1* and *ABCG1* in M-MØ and GM-MØ incubated with acetyl-LDL (ac-LDL) or oxidized-LDL (ox-LDL), both ligands of scavenger receptors, or native LDL, which can also induce macrophage cholesterol accumulation by fluid pinocytosis^42^. Adding equal concentrations of LDL (25 µg/mL) to the culture media, macrophage cholesterol contents were induced efficiently by ac-LDL, and modestly by ox-LDL and native LDL in either macrophage subtype (Fig. 5A). As predicted by the higher levels of scavenger receptor expression in M-MØ (see Fig. 1), exposure to ac-LDL induced cholesterol accumulation by about twofold in the M-MØ compared to GM-MØ. Overall, irrespective of the LDL type used for cholesterol-loading, macrophages overexpressed both ABC mRNAs in a similar manner, and quantitatively proportional to the degree of loading. Yet, of the 2 ABC transporters, *ABCG1* expression was stronger in both CSF foam cell subtypes. Accordingly, a robust loading of M-MØ with ac-LDL increased the cellular cholesterol content about fivefold, which was related to about fivefold increase in ABCA1 expression and a 40-fold increase in *ABCG1* expression (Fig. 5B,C). Analysis of the expression of transcription factors indicated that M-MØ expressed significantly higher basal levels of *LXRA* and *PPARG* than the GM-MØ subtype and in response to ac-LDL treatment the expression of these transcription factors significantly increased only in M-MØ foam cells (Fig. 5D,E). In contrast, the expression of *PPARG* was similar between the 2 non-loaded macrophage subtypes and was increased by cholesterol loading only in GM-MØ (Fig. 5F).

### M-MØ foam cells are functionally competent in cholesterol efflux

**Figure 6:**
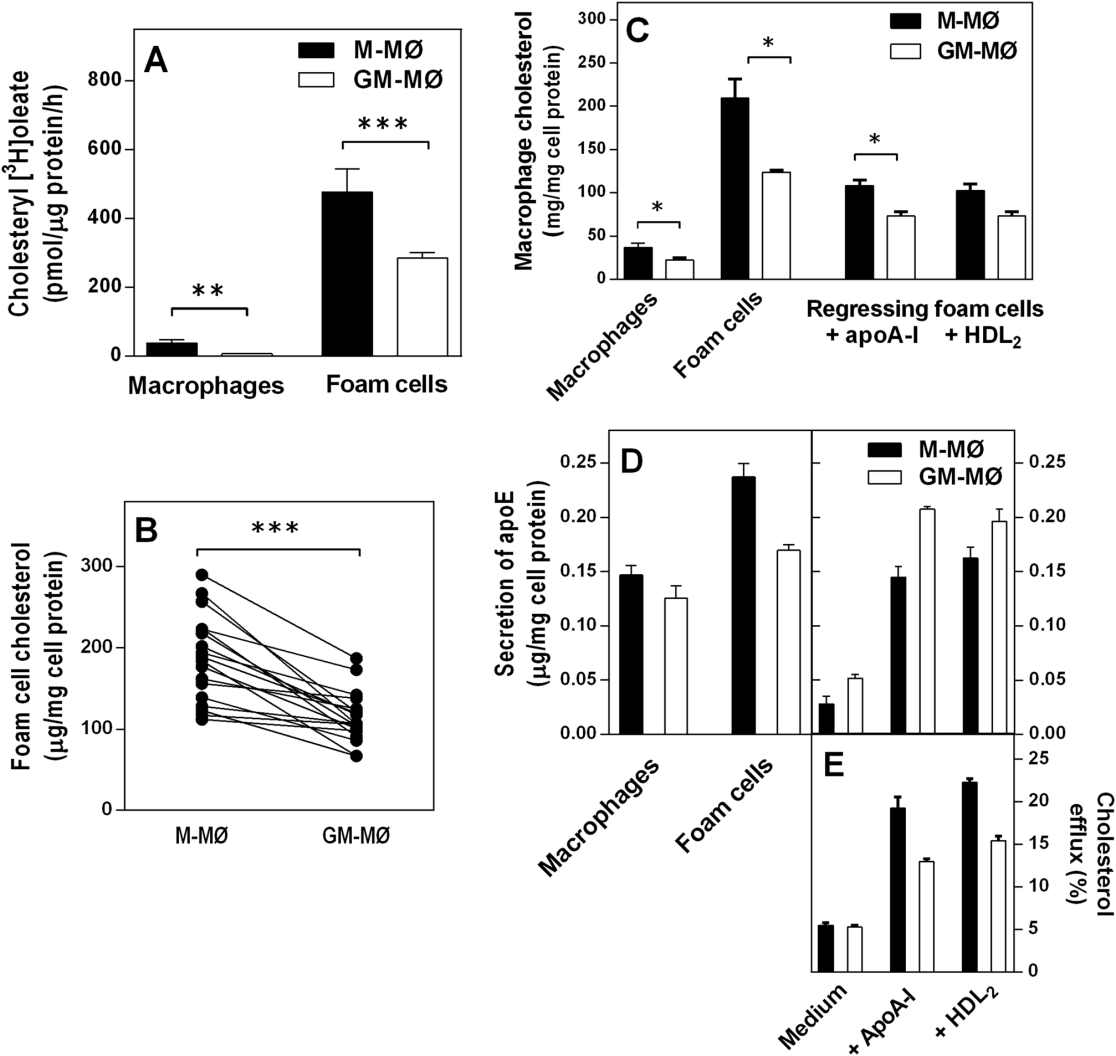
Cholesterol esterification activity, apoE secretion, and cholesterol efflux in M-MØ and GM-MØ foam cells. (**A**) ACAT1-mediated esterification activity was measured as the rate of [^3^H]oleate incorporation into cholesteryl esters in M-MØ and GM-MØ which were incubated in the absence (Macrophages) or presence (Foam cells) of acetyl-LDL, after which the cells were incubated with [^3^H]oleic acid-BSA complexes. Cellular lipids were extracted and [^3^H]-activity was determined. (**B**) Cholesterol contents in the foam cells of each CSF subtype. (**C**) Cholesterol contents in M-MØ and GM-MØ before (Macrophages) and after incubation with acetyl-LDL (Foam cells), and after cholesterol efflux to apoA-I (10 µg/mL) and HDL_2_ (25 µg/mL) (Regressing foam cells). (**D**) Macrophages were loaded with cholesterol and cholesterol efflux was induced by adding apoA-I or HDL_2_ to the incubation medium, and the concentrations of apoE in the media were determined by ELISA. (**E**) Cholesterol efflux from [^3^H]CE-labeled macrophage foam cells of either subtype was evaluated by measuring the macrophage-derived [^3^H]-radioactivity in media when the cells were incubated with medium alone, apoA-I or HDL_2_. Fractional efflux was calculated as [dpm medium/(dpm cells + dpm medium)] × 100. Data are presented as means ± SEM of 9 (**A**); 17 (**B**); 2 (**C**); 6 (**D**); and 6 (**E**) individual blood donors and triplicate incubations for each studied condition. **p < 0.01 and ***p < 0.001 by Student's t-test for paired samples (**A**,**B**); *p < 0.05 by Wilcoxon Signed-Rank test for paired samples (triplicate wells per assay) (**C**); *p < 0.05 by Student's t-test for paired samples (**D**); *p < 0.05 by Student's t-test for paired samples (**E**).

Next, we determined the efficiency of the 2 macrophage foam cell subtypes to esterify internalized cholesterol and release their cholesterol cargo in response to extracellular cholesterol acceptors (Fig. 6). Since cholesterol esterification via ACAT1 is the main mechanism available to attenuate cytotoxic free cholesterol accumulation and deposit as cholesterol fatty acyl esters in lipid droplets, we evaluated ACAT1 activity in both CSF macrophage subtypes. For this, the rates of [^3^H]oleate incorporation into cholesteryl esters were measured in M-MØ and GM-MØ in the absence or presence of ac-LDL. Consistent with higher ACAT1 expression in M-MØ (see Fig. 1), these cells also displayed greater efficiency for cholesterol esterification, both in the non-loaded and cholesterol-loaded phenotypes, as compared to the respective GM-MØ (Fig. 6A). The data (Fig. 6B) accord with their higher capacity for cholesterol uptake reported earlier^34^. The cholesterol content of M-MØ and GM-MØ foam cells were also quantified after incubation with apoA-I or HDL_2_ which stimulate ABCA1- or ABCG1-dependent cholesterol efflux pathways, respectively^8^ (see the experimental outline in Supplementary Fig. S1). As shown in Fig. 6C, macrophage cholesterol depletion was efficiently induced by incubating either CSF foam cell subtype with each cholesterol acceptor, yet the residual cellular cholesterol cargo remained higher in the regressing M-MØ foam cells.

For the fact that human macrophages do not secrete apoA-I, yet they can secrete small amounts of apoE^43^ as an alternative ligand to stimulate ABCA1-mediated cholesterol efflux^29^, we analyzed apoE contents in the culture media and determined the rates of apoE secretion from non-loaded, cholesterol-loaded, and in regressing macrophages of both CSF subtypes (Fig. 6D). In accordance to earlier reports^44,45^, cholesterol loading increased apoE secretion both in M-MØ and GM-MØ whereas the non-loaded and cholesterol-loaded M-MØ secreted greater amounts of apoE compared to the respective GM-MØ (left panel). This reflects both stimulatory as well as inhibitory effects on apoE secretion reported in human macrophages exposed either to M-CSF or to GM-CSF^46,47^. Next, the culture media were replaced and the foam cells of either CSF subtype were incubated for 18 h in fresh medium in the absence or presence of apoA-I or HDL_2_. Quantitation of secreted apoE in the culture media (right panel) showed low secretion from foam cell macrophages incubated in medium alone. In contrast and in line with previous reports^48,49^, supplementing the media with apoA-I or HDL_2_ significantly increased apoE secretion, which was more enhanced in GM-MØ. The more robust modulatory effects of cholesterol loading and cholesterol efflux on M-MØ indicate that the sterol-mediated regulation of apoE secretion is more tightly coordinated in the M-MØ rather than in GM-MØ. Finally, the cholesterol efflux capacity of [^3^H]CE-labeled macrophage foam cells of either subtype was evaluated (Fig. 6E). Notably, the addition of either apoA-I or HDL_2_ to the macrophage foam cell cultures significantly induced more efficient cholesterol efflux from the macrophages that had been polarized in M-CSF. In summary, these data indicate that greater residual cholesterol cargo found in the regressing M-MØ foam cells (Fig. 6C) was due to their higher cholesterol uptake and esterification properties and does not derive from any compromised cholesterol removal capacity via the ABCA1 or ABCG1 pathways.

### GM-MØ support greater T cell proliferation than M-MØ in the presence of PHA

**Figure 7:**
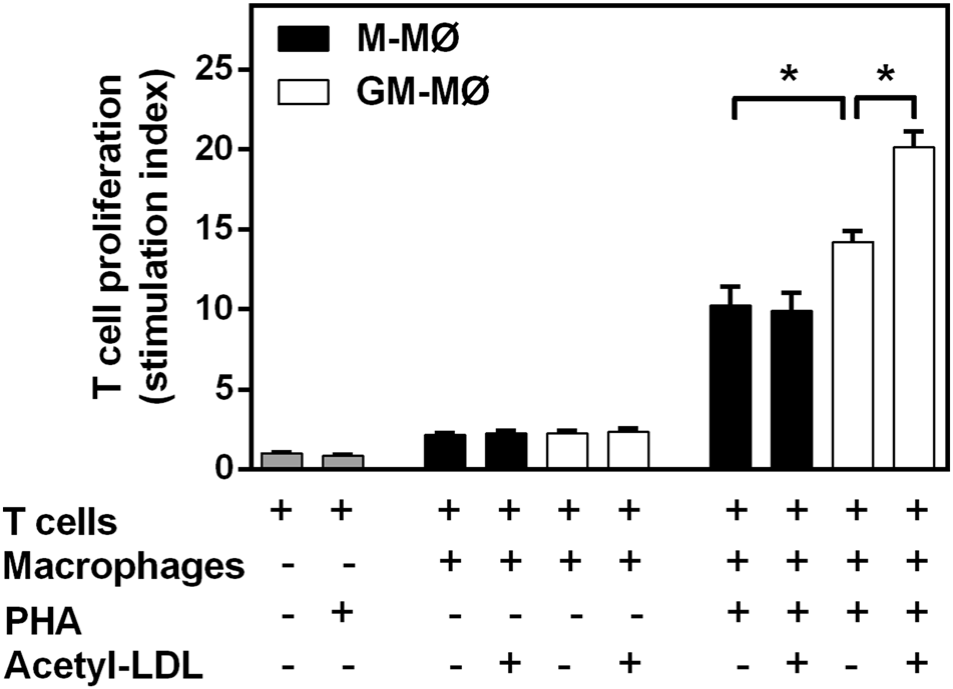
Effect of lipid-loading on the capability of M-MØ and GM-MØ to support T cell proliferation. For each experiment, M-MØ and GM-MØ were prepared from a buffy coat derived from a single human donor. The macrophages were incubated in the absence or presence of acetyl-LDL (25 µg/mL) for 24 h in serum-free DMEM, after which the cells were washed, autologous T cells were added and co-cultured in the presence or absence of 10 µg/mL phytohemagglutinin (PHA). As a control, the T cells were cultured alone (grey bars). After 4 days, the T cell numbers in the medium were counted. T cell proliferation was expressed as a stimulation index defined as a ratio obtained by dividing the number of viable T cells in the presence of macrophages by the number of viable T cells in the absence of macrophages. Data (means ± SEM) are derived from triplicate wells and correspond to 3 monocyte donors. *p < 0.05 by Wilcoxon Signed-Rank test for paired samples.

Finally, to get further insight into other key functional feature of the CSF-macrophage foam cells, we evaluated the effect of cholesterol loading on the capacity of each CSF macrophage subtype to interact with surrounding cells. Since both macrophages and T cells coexist in atherosclerotic lesions and play a major role in atherosclerosis progression, we cocultured GM- and M-MØ with T cells isolated from the respective donors and investigated if cholesterol loading of CSF-polarized macrophages also elicited distinct macrophage immune responses affecting proliferation of neighboring T cells via cell–cell interactions. For this purpose, autologous T cells were co-cultured with non-loaded or cholesterol-loaded macrophages of either CSF subtype in the presence of the mitogen phytohemagglutinin (PHA) to induce T cell proliferation. As shown in Fig. 7, in the presence, but not absence of PHA, co-culture with either non-loaded macrophage subtype was able to induce T cell proliferation, yet, the GM-MØ were more potent accessory cells compared to M-MØ. Moreover, cholesterol loading of GM-MØ, but not M-MØ, increased their ability to induce T cell responses in autologous cultures, so enhancing T cell proliferation, which has been implicated in the growth of atherosclerotic plaque in mice^50^. Regarding the ability of macrophages to support T cell survival, no significant differences were observed between non-loaded or cholesterol-loaded M-MØ or GM-MØ (not shown).

## Discussion

M-CSF is a cytokine constitutively expressed and ubiquitously produced by many cell types, while GM-CSF is mainly produced by activated leukocytes and synthesized in large amounts under various inflammatory conditions^51^. Thus, differentiation of macrophages with M-CSF or GM-CSF has been described to prime the cells toward an anti-inflammatory or pro-inflammatory phenotype, respectively. However, several reports indicate that these polarization pathways regarding inflammatory activation have not been fully reached in the CSF-differentiated macrophages^26,51^. Moreover, lack of consensus on the differentiation protocols in vitro and the available nomenclature to clearly define the generated macrophage subpopulations have led to many discrepancies and calls for the establishment of uniform standards in experimental studies^52^. In this regard, the differentiation of human blood-derived monocytes into macrophages in the presence of serum typically generates heterogeneous macrophage populations^53^ since serum preparations contain variable concentrations of cytokines and growth factors, among them the M-CSF and the GM-CSF, and also an array of potential activators of signaling pathways in macrophages, which affect both their phenotype and function. The use of a culture medium of complex composition can lead to confounding and as well as potentially uncontrollable effects due to a potential switch in macrophage polarization or due to induction of macrophage cholesterol efflux by apoA-I-containing particles in the medium^54^. Moreover, since the commercially available serum preparations are derived from various animal species, from individual human subjects or various human subpopulations without standardization, much compositional lot-to-lot variation exists^55^. Finally, sera also contain variable concentrations of TGF-β, a strong inhibitor of macrophage activation^56^. Based on the above knowledge, the present study utilized strict serum-free cell culture conditions consisting of “macrophage serum-free medium” with a given concentration of either M-CSF or GM-CSF and in the absence of any added cytokines. This allowed us to avoid an uncontrollable heterogeneity of CSF-polarized subpopulations of human macrophages, and to identify CSF-sensitive genes. Importantly, the polarized macrophages exhibited memory for CSF-specific antigen expression patterns (CD68^+^/CD14^+^ and CD68^+^/CD14^−^) previously verified under more complex cell culture conditions^17,19^, which resembled those existing in human atherosclerotic plaques^19^. Moreover, the present data extend earlier observations by demonstrating that an increased foam cell-forming potential of M-CSF-polarized macrophages exposed to native or modified LDL^19,34^ does not require any additional serum-derived components.

Consistent with earlier observations by Kruth and coworkers^19^, we found that the presence of either CSF during the monocyte-to-macrophage differentiation period distinctly affected the gene profiles, when studied in fully mature macrophages. Interestingly, our findings regarding the expression of lipid metabolic genes concur with that study^19^ for *CCL2* and *ABCG1*, whilst other data such as increased levels of PPARγ and LXR observed for GM-MØ did not. Overall, both the foam cell-forming capacity and pro-inflammatory potential of M-CSF-treated macrophages was stronger than that of GM-CSF-treated macrophages. However, cholesterol loading modified the M-CSF-dependent polarization of a set of genes regulating macrophage inflammatory responses and also resulted in foam cells with a milder pro-inflammatory state. Of interest, in vivo, increased cholesterol efflux from tissues to plasma HDL has been reported in M-CSF-treated hypercholesterolemic WHHL rabbits^34^. In line with this observation, we found that despite the more abundant scavenger receptor-mediated uptake of acetylated-LDL particles by M-CSF-polarized foam cells, these cells were highly efficient in counteracting intracellular free cholesterol accumulation by enhancing the ACAT1-mediated cholesterol esterification^57^ and by increasing the rate of ABC transporter-mediated cholesterol efflux. Therefore, the abundant uptake of LDL by M-MØ with simultaneous yet unaffected cholesterol efflux was responsible for the high lipid load of these cells at any stage. These distinct responses of CSF-polarized macrophage foam cells revealed that cholesterol loading may alter the expression of specific M-CSF polarization-induced genes towards a less atheroinflammatory phenotype, thereby revealing that these foam cells had gained atheroprotective features.

Attenuation of the CSF-dependent phenotypic divergence of macrophages based on cholesterol loading was exemplified by the convergence in the expression of *ABCG1*, a cholesterol efflux transporter also possessing anti-inflammatory effects demonstrated in vitro and in mice in vivo^13,31,58–60^, and in the expression of *CCL2* as an early component of the pro-inflammatory response in atherosclerosis^32,61^. In fact, the M-CSF/GM-CSF-ratios of *ABCG1* and *CCL2* mRNA expression in the non-loaded macrophages tended to approach value 1 (i.e. unchanged) in the foam cells (Supplementary Fig. S5). To our knowledge, the present study exploiting a well-defined serum-free cell culture system provides the first evidence that cholesterol loading can override the differential inflammatory profiles in CSF-polarized macrophages by reprogramming the gene expression levels of both *ABCG1* and *CCL2* towards an anti-inflammatory phenotype.

Although macrophage cholesterol accumulation is intuitively linked to accelerated atherogenesis, we have observed that the conversion of cultured human macrophages into foam cells suppresses their proinflammatory responses to M1-polarizing factors^62^. Moreover, it has been shown in vivo that the foam cells in apoE knock-out mice express a beneficial pro-fibrotic phenotype, which tends to restore the stability of atherosclerotic plaques^63^. This suggests that the foamy transformation of some macrophage subtypes may represent beneficial features in maintaining vascular homeostasis, at least in macrophage-rich early lesions. To test this notion further, we activated macrophages with LPS, which has been widely used as a potent stimulatory agent for transcription of several genes encoding proinflammatory mediators in macrophages and thereby inducing an inflammatory state. In vivo studies in mice have shown that while M-CSF primes macrophages for LPS-dependent cytokine induction such as IL-1β, TNF-α and IFN-γ^64^, treatment with GM-CSF alters the LPS-induced cytokine expression and decreases the synthesis of proinflammatory cytokines thereby increasing their anti-inflammatory properties^65^. Notably, we found that cholesterol loading, by suppressing LPS-dependent upregulation of the expression of *IL1B*, *TNFA*, and *CXCL8*, reduced the pro-inflammatory gene expression profile in both macrophage subtypes. Similar attenuation of pro-inflammatory response in LPS-activated macrophages, as found in the present study, has been observed in mouse peritoneal macrophages treated with ox-LDL^66^. Under such conditions, the transcriptional machinery of LPS-induced pro-inflammatory genes was inhibited, suggesting that ox-LDL loading targets specific TLR-induced mechanisms required for the upregulated expression of a subset of pro-inflammatory genes^66^. In line with these findings, other reports have indicated that macrophages cholesterol accumulation does not lead to an inevitable pro-inflammatory phenotype, but associate with suppression of pro-inflammatory gene expression instead^24,62,67^. Importantly, a mechanistic link between cholesterol accumulation and suppression of inflammation in macrophages has been suggested due to a regulated accumulation of desmosterol in cells as the last intermediate in the pathway of cholesterol biosynthesis^68^. On the whole, the above findings demonstrate that macrophage foam cells initiate a transcriptional program that prevents inflammatory responses during cholesterol loading, as also shown in vitro in the present study.

LXR-dependent signaling modulates the subcellular responses elicited by macrophage cholesterol efflux^35^, which is partly controlled at the transcriptional level by nuclear receptors in response to sterol loading-induced ABCA1 transporter and stimulation of lipid efflux to extracellular acceptors^69^. In human macrophage foam cells, both ABCA1 and ABCG1 are induced via oxysterol-dependent LXR activation, but also differentially regulated by PPAR-agonists. Indeed, ABCA1 is regulated by both PPARα and PPARγ, while ABCG1 is regulated by PPARγ alone^35^. Moreover, the concerted expression of ABCA1 and ABCG1 in THP-1 macrophages likely relies on a PPARγ-LXR-dependent pathway^70^. Of interest to the present study, M-CSF has been shown to rapidly repress (within 6 h) the expression of ABCG1 in human macrophages^71^. Notably, we found that the sharp increase of ABCG1 expression in response to ac-LDL, ox-LDL, or native LDL in the M-MØ subtype was paralleled by overexpression of *LXRA* and *PPARA*, but not that of *PPARG*. These M-MØ responses contrasted those of the GM-MØ subtype, in which ABCG1 was only slightly increased while PPARG was exclusively upregulated. Studies in THP-1 cells treated with LXR-agonists have shown that ABCG1 is very sensitive to LXR-dependent induction^72^, a finding compatible with our results on the M-MØ subtype. Indeed, our data do not support an involvement of PPARγ in *ABCG1* induction, but rather suggest a specific LXRα gain-of-function activity during cholesterol loading in M-CSF-polarized macrophages. Importantly, LXRs are thought to reciprocally regulate genes linked to lipid efflux and inflammation^73^. Therefore, while mouse macrophages induce *ABCA1* expression in response to LXR ligands, genes related to inflammation, such as *CCL2*, are being repressed. Previously, a reduced down-stream expression of *CCL2* has been linked to LXR activation^74^. Conversely, more recent studies have found that cholesterol efflux can remodel the lipidome of foam cells^75^. A regulated increase of desmosterol underlies the activation of LXR target genes and thereby discloses a mechanistic link between cholesterol accumulation and suppression of macrophage inflammation^68^. On the contrary, in the brain of Abcg1/Abcg4-deficient mice, several sterol intermediates of the cholesterol biosynthetic pathway, desmosterol among them, were increased by two- to threefold and associated with increased expression of LXR target genes, including *ABCA1*, and increased secretion of apoE^76^. Altogether, the present data show that expression of both *ABCG1* and *CCL2* appears to be regulated in an opposite pattern by LXRα in M-CSF-polarized foam cells. Furthermore, our results demonstrate that, provided foam cells are formed, the degree of ABC transporter mRNA upregulation is associated with total cholesterol load in the macrophages and independent of the type of LDL used for cholesterol loading as well as the CSF subtype of macrophages.

Earlier, mouse macrophages were found to be more competent accessory cells in supporting T cell proliferation when differentiated with GM-CSF rather than with M-CSF^37^. Complementary to those observations, we observed that the GM-MØ were acting as more potent accessory cells compared to the M-MØ. Moreover, in agreement with Munn et al.^77^, the macrophages differentiated under the influence of M-CSF did acquire the ability to suppress T cell proliferation. Since this accessory effect was specific to the foam cells of the GM-MØ subtype, the overall findings suggest that the intrinsically divergent properties elicited by either CSF in macrophages for regulating T cell proliferation were not affected by the cholesterol loading.

A potential limitation of the present work is that it focused on the mRNA expression of selected genes, which does not always reflect the standard dichotomy of the translational process into a functional protein. Nevertheless, our study demonstrates distinct metabolic responses to cellular cholesterol loading and cholesterol efflux in M-CSF- and GM-CSF-differentiated human macrophages in vitro. Importantly, the effects of cholesterol loading on the studied genes were segregated into those remaining as quiescent markers of the CSF-polarized cells, e.g. the CD antigens, and those altering their expression in the foam cells, notably *ABCG1* and *CCL2*. Thus, while the CSFs trigger monocyte transition into macrophages and also condition the alternatively generated macrophages to potentially distinct atheroinflammatory cytokine-mediated responses, the subsequent process of foam cell formation, i.e., in the absence of any accessory pro-inflammatory stimuli, evidently attenuated their ultimate pro-inflammatory potential. This information is of crucial importance as the primary actions of macrophages in atherosclerotic lesions relate to the regulation of two interrelated processes, namely cholesteryl ester storage and inflammation. Collectively, our results suggest that irrespective of the type of CSF-polarization, the transformation of lesional macrophages into foam cells not only protects the cells from the toxicity of free cholesterol but also reduces their pro-inflammatory actions in the atherosclerotic lesions. Despite our efforts, however, the present cell culture method remains an incomplete surrogate of a model in the complex and ever-changing extracellular microenvironment in which the lesional macrophages reside during atherogenesis. But even so, our system offers a delicate framework for future studies, in which the effects of M-CSF and GM-CSF on macrophage polarization and biology, in general, can be studied in a more complex and well-defined culture media using time-varying variables, i.e. covering the various stages along the long path from a monocyte to a macrophage foam cell, and beyond.

## Methods

### Human plasma lipoproteins

Plasma derived from healthy volunteers was supplied by the Finnish Red Cross Blood Service (Helsinki, Finland). Human LDL (1.019–1.050 g/mL) and HDL_2_ (1.063–1.125 g/mL) were isolated by sequential ultracentrifugation using KBr for density adjustments, according to standard techniques^78^. To prevent lipoprotein oxidation, all steps were performed in the presence of 3 mM EDTA. Lipoprotein concentrations are expressed for their protein content^79^. LDL was acetylated (acetyl-LDL) with acetic anhydride, as described^80^. To generate oxidized LDL (ox-LDL), native LDL (1 mg protein/mL) was incubated with 10 µM CuSO_4_ at 37 °C for 18 h, following the method originally described by Steinbrecher and co-workers^81^. At the end of the incubation, the oxidation reactions were terminated by adding EDTA (final concentration of 100 µM) to the incubation mixture, and by cooling the sample on ice. The various modified LDL preparations were derived from a single LDL preparation (Supplementary Table S1). The preparations were endotoxin-free when assayed by the Limulus Amebocyte Lysate kit according to the manufacturer’s instructions (Cambrex Bio Science Walkersville Inc. MD, USA). All lipoprotein preparations were stored in dark at 4 °C until use.

### Primary monocyte-macrophage cultures

The experimental outline is shown in Supplementary Fig. S1. Human monocytes were differentiated into macrophages in the presence of CSF^62^, with modifications relevant to this particular study. Buffy coats from volunteer healthy donors were provided by the Finnish Red Cross Blood Transfusion Service, Helsinki, Finland (supplied under permits 45/2012 and 2/2014), and they were layered over Ficoll-Paque and centrifuged at 800×*g* for 30 min. As previously described^62^, the peripheral blood mononuclear cells (PBMC) were collected, sedimented, and washed 3 times with PBS to remove contaminating platelets, and finally suspended in Dulbecco’s Modified Eagle Medium (DMEM, Lonza, Verviers, Belgium). The washed PBMCs were transferred onto 24-well plates (1.5 million cells/well) and allowed to adhere for 1 h, after which the medium was removed and replaced by adding macrophage serum-free medium (SFM, Invitrogen, Carlsbad, CA) containing 50 ng/mL of M-CSF or 10 ng/mL of GM-CSF (both from Biosite Ltd, UK). The cells were then cultured for 6 days in SFM, and, during this period the CSF-containing medium was replaced every 2–3 days in an attempt to keep the concentrations of the CSFs as constant as possible. After 6 days of culture, the cells were ready for experimentation as judged by their phenotypical (morphological) conversion into macrophages^82^. Macrophage maturation was also confirmed by immunostaining of the cells for the common macrophage marker CD68. Altogether, we investigated macrophages derived from a total of 75 human donors.

### Immunostaining of cultured monocyte-derived macrophages

Detection of the common macrophage markers CD14 and CD68 was performed as described earlier^19^. In brief, cells were first rinsed 3 times with phosphate-buffered saline (PBS, Lonza, Verviers, Belgium) and then fixed in culture dishes with methanol (VWR International, West Chester, PA) at − 20 °C for 5 min, and finally blocked with PBS containing 10% normal human serum (Pel-Freez, Milwaukee, WI) for 20 min. The cells were then incubated for 60 min in the presence of 2 µg/mL of primary antibody, which was goat anti-human CD68 or mouse anti-human CD14 (both from Santa Cruz Biotechnology, Santa Cruz, CA) or mouse anti-human mature macrophage, clone 25F9 (from BMA Biomedicals, Augst, Switzerland). Non-specific goat and mouse IgGs (Santa Cruz Biotechnology) were used as negative controls. After washes, 5 µg/mL of a biotinylated secondary antibody, which was either horse anti-goat or goat anti-mouse IgG (both from Vector Laboratories, Burlingame, CA) was added and incubated for 45 min, and then followed by 15 min incubation with 10 µg/mL of avidin-labeled Alexa 488 or Alexa 594 (Molecular Probes, Eugene, OR). The cells were rinsed 3 times in PBS after each incubation at room temperature.

### Flow cytometry

Analysis of cell surface antigens was performed as described^83^, with slight modifications. Briefly, the cells were detached from culture dishes with Accutase solution (Chemicon) and resuspended in prechilled fluorescence-activated cell sorter buffer (PBS with 0.5% BSA and 0.025% NaN_3_), and fixed with 2% paraformaldehyde/PBS. After 2 washes at room temperature, the cells were blocked with 5% FBS/PBS, permeabilized with saponin, and stained for CD68, CD11c and CCR7 by using fluorescent-labeled antibodies for 30 min on ice. Finally, following 2 washes at room temperature, the cells were analyzed by using an LSR II flow cytometer (BD Biosciences, San Jose, CA). Isotype-matched mouse IgGs were used as negative controls.

### Generation of macrophage foam cells and measurement of their cellular cholesterol contents

Monocyte-derived macrophages of each CSF subtype were incubated in DMEM containing 25 µg/mL of acetyl-LDL for 24 h to generate macrophage foam cells, as described previously^82^. For the measurement of cellular cholesterol contents, the cells were detached from the culture dishes, sonicated, and the protein levels in the cellular lysates were then measured. Cellular lipids were extracted with hexane–isopropanol (3:2; v/v). Free cholesterol and cholesteryl esters in the lipid extracts were separated by high-performance thin-layer chromatography (HP-TLC) followed by quantitative analysis with an automatic plate scanner (CAMAG TLC). Total cholesterol content was calculated as the sum of free and esterified cholesterol fraction in each sample. In other experiments, the respective CSF was added to the cholesterol loading medium at the same concentration as used for monocyte differentiation, or the macrophages were loaded with cholesterol by incubation for 24 h with oxidized-LDL or native LDL (25 µg/mL, each).

### Cholesterol efflux

Macrophage foam cells of either CSF subtype were washed with PBS, after which fresh DMEM was added. The DMEM contained either purified human lipid-free apoA-I (10 µg/mL; kindly provided by Dr. Peter Lerch of the Swiss Red Cross), HDL_2_ (25 µg/mL), or human plasma (2.5%, vol/vol) and cholesterol efflux was evaluated, as previously described^83^. In brief, after incubation for 18 h with the various cholesterol acceptors, the media were collected, centrifuged at 300*g* for 10 min to remove cellular debris, after which the [^3^H]-radioactivity in the medium and in the washed cells was determined by scintillation counting. The fractional cholesterol efflux (in %) was calculated as follows: dpm in medium/(dpm in cells + dpm in medium) × 100^83^. Cholesterol efflux into the incubation medium in the absence of any added extracellular acceptor was subtracted from the efflux values obtained in the presence of acceptors. The concentration of apoE in cell-free media was determined by ELISA, as described previously^84^.

### Analysis of gene expression

The mRNA expression levels of selected genes encoding proteins related to cholesterol balance and inflammation were analyzed by quantitative RT-PCR in non-loaded macrophages and cholesterol-loaded macrophage foam cells of each CSF subtype. In some experiments, gene expression was analyzed in activated macrophages after their incubation for 3 h with LPS (100 ng/mL). Real-time PCR was performed as previously described^85^. Accordingly, total cellular RNA was isolated using the RNeasy Plus Kit with the removal of genomic DNA according to the manufacturer’s instructions (Qiagen, Valencia, CA). Nucleic acid concentrations were determined by NanoDrop ND-1000 spectrophotometer (NanoDrop Technologies, Wilmington, DE). The RNA from each sample was then converted to cDNA using Moloney-Murine Leukaemia Virus (M-MLV) reverse transcriptase and random hexamers (both from Promega, Wilmington, WI, USA) for 55 min at 48 °C. For PCR, the samples were amplified in duplicate with ABI Prism 7500 Sequence Detector System using TaqMan Universal or Power SYBR Green PCR Master Mix (all from PE Applied Biosystems) with the program settings: 10 min pre-incubation at 95 °C followed by 40 amplification cycles consisting of 15-s denaturation at 94 °C and primer annealing and polymerase extension for 60 s at 60 °C. Oligonucleotide sequences of the quantitative PCR primers and 5′-FAM labeled hydrolysis probes with non-fluorescent quencher are shown in Supplementary Table S2. The data were normalized relative to an endogenous control glyceraldehyde-3 phosphate dehydrogenase (GAPDH) by applying the delta-deltaCt algorithm.

### Evaluation of T cell proliferation

T cells were purified from buffy coats using RosetteSep human T cell enrichment cocktail according to the manufacturer's instructions (StemCell Technologies, Vancouver, Canada). To remove adhered monocytes, the obtained T cells were purified further by incubating them for 1 h at 37 °C in serum-free DMEM. The purified T cells were cultured in serum-free OpTmizer medium (Invitrogen) or cryopreserved until used. M-MØ and GM-MØ derived from the same buffy coats were incubated in the absence or presence of acetyl-LDL (25 µg/mL) for 24 h in serum-free DMEM. The cells were extensively washed and the previously cryopreserved autologous T cells (125,000 to 500,000 cells/mL; T cell: macrophage ratio ranging from 0.5:1 to 1.5:1) were added and co-cultured in serum-free OpTmizer in the presence or absence of 10 µg/mL phytohemagglutinin (PHA: Sigma). After 4 days, the plates were swirled, pipetted vigorously to disrupt any clump, and the T cell numbers in the medium were counted using a nucleocounter (NC-200 Cell Counter ChemoMetec A/S). As a control, macrophages and T cells were cultured alone. Under our experimental conditions, only negligible (less than 5000 cells) numbers of macrophages were found in the medium. The T cell proliferation is expressed as a stimulation index (SI), which was calculated by dividing the numbers of viable cells in the medium in the presence of macrophages by the number of viable cells in the medium containing T cells only.

### Statistics

Differences in continuous variables from 2 experimental groups were assessed by using Wilcoxon Signed-Rank test or unpaired-samples t-test, when appropriate. Statistical significance was set at p < 0.05. One-way ANOVA with Bonferroni's Multiple Comparison Test was used to compare data from 3 or more groups. GraphPad Prism 5.0 software (GraphPad, San Diego, CA) was used for all statistical analyses.

## Supplementary Information

Supplementary Information.

## Data Availability

All data generated or analyzed during this study are included in this article and its Supplementary Information. No datasets were generated or analyzed during the present study.
