# Supplementary material for: Cholesterol loading suppresses the atheroinflammatory gene polarization of human macrophages induced by colony stimulating factors
Source: Sci Rep. 2021 Mar 1;11:4923. doi: 10.1038/s41598-021-84249-y (PMC7921113; doi:10.1038/s41598-021-84249-y)
Supplement: Supplementary file 1 — Supplementary Information. [file 41598_2021_84249_MOESM1_ESM.pdf]

SUPPLEMENTARY MATERIAL

Cholesterol Loading Suppresses the Atheroinflammatory Gene Polarization of Human Macrophages Induced by Colony Stimulating Factors

Jani Lappalainen, Nicolas Yeung, Su D. Nguyen, Matti Jauhiainen, Petri T. Kovanen, Miriam Lee-Rueckert

Supplementary Figures S1 – S5

Supplementary Tables S1 and S2

Supplementary Figure S1

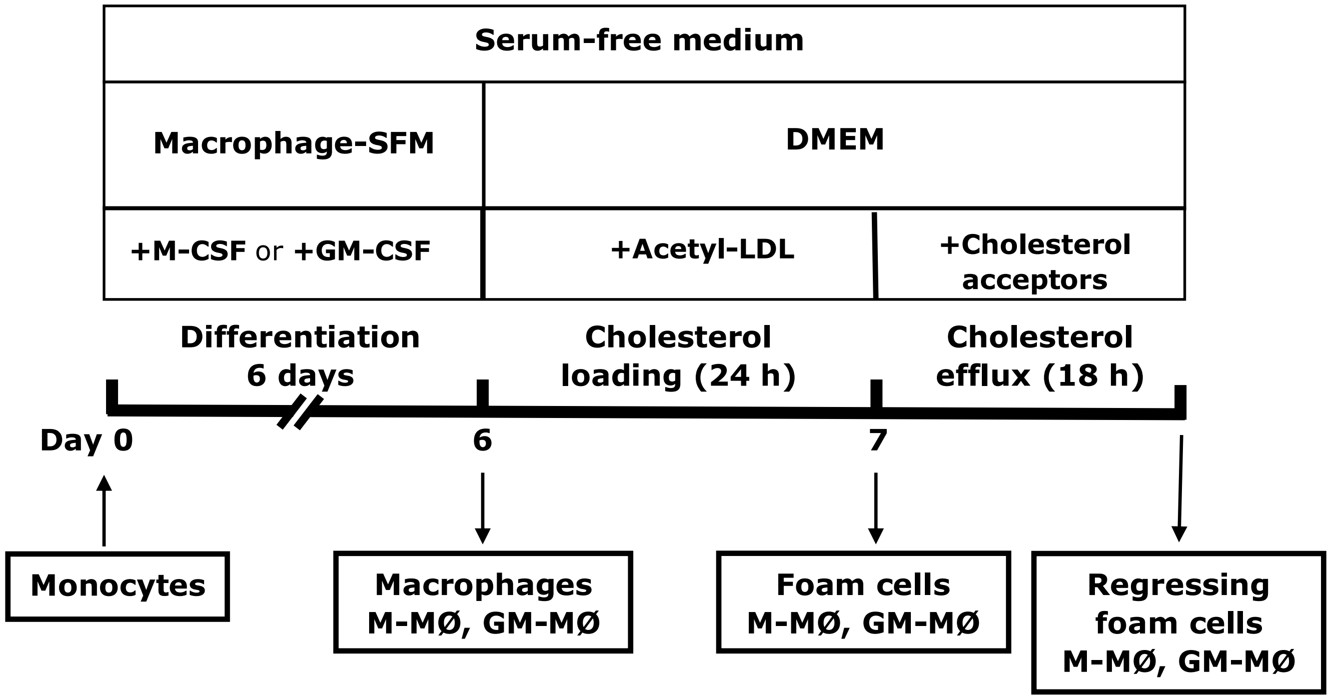

Figure S1. ***Experimental outline for cell culture***
Human monocytes were differentiated for 6 days in macrophage serum-free medium containing M-CSF (50 ng/mL) or GM-CSF (10 ng/mL) to generate "Macrophages" of the M-MØ and GM-MØ subtypes, respectively. Both macrophage subtypes were cholesterol-loaded (Foam cells) by subsequent incubation for 24 h in DMEM containing acetyl-LDL (25 µg/mL). Thereafter, cellular cholesterol efflux was stimulated by incubation of the foam cells for 18 h in DMEM containing various cholesterol acceptors (Regressing foam cells). After each experimental step, the cells and the media were subjected to various analyses.

Supplementary Figure S2

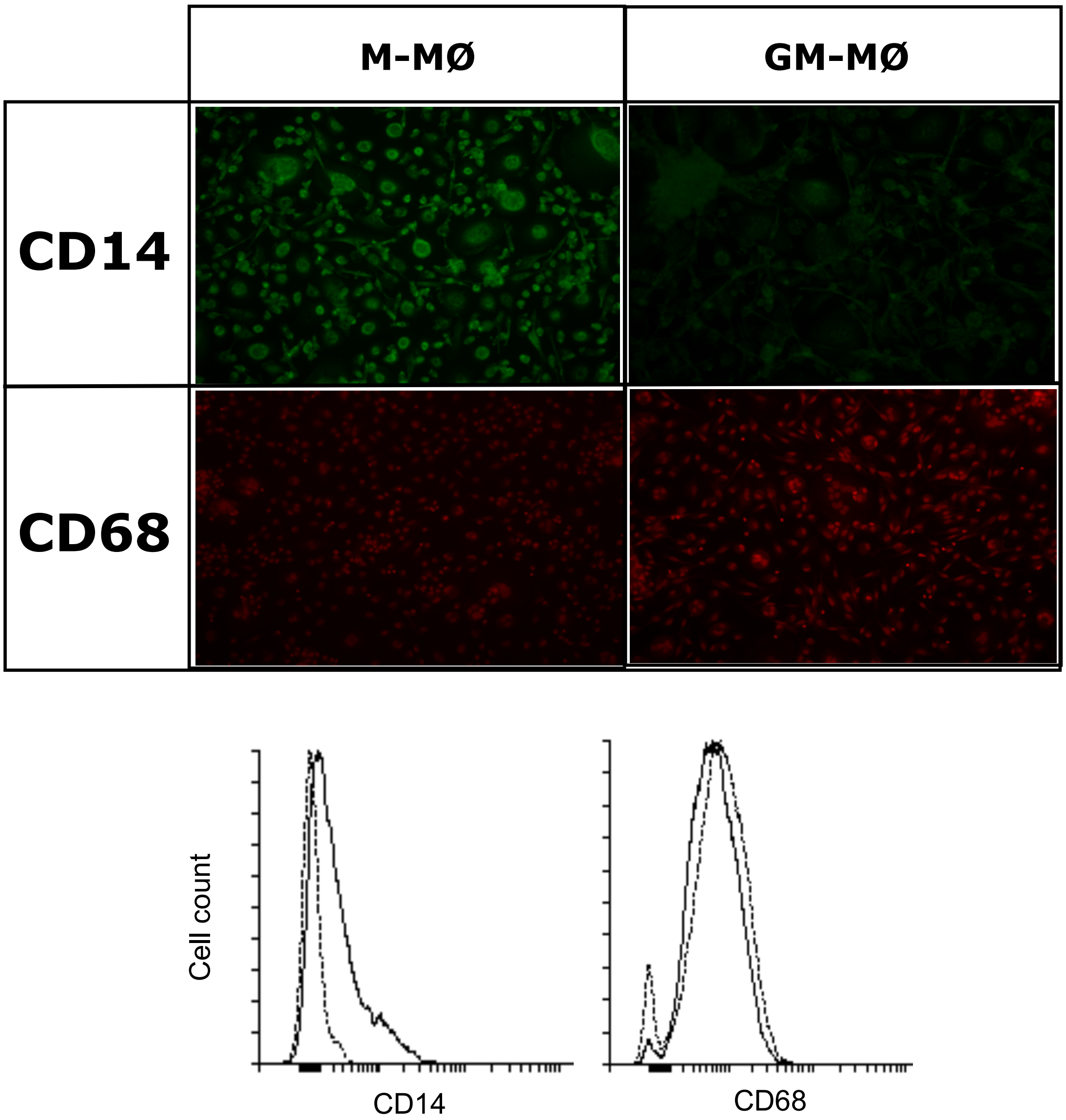

Figure legend S2. ***Detection of macrophage surface markers in monocytes differentiated with M-CSF or GM-CSF***

Peripheral blood monocytes that were allowed to adhere for 2 h on plastic dishes appeared as round with small diameter (≈ 9-15 μm) and scant cytoplasm (not shown). When grown in serum-free medium without any added growth factor, these non-differentiated cells died after 5 days of incubation. **Top panel**: Monocytes differentiated with M-CSF were typically elongated and ”sausage”-shape with numerous vacuoles whereas those differentiated with GM-CSF displayed a typical "fried-egg"-like morphology (not shown). The cells were immunostained for the macrophage markers CD14 and CD68 and representative images were acquired. **Bottom panel**: The cells were also analyzed by flow cytometry and the data is presented as overlaid histograms of antigen expression intensity for M-MØ (solid line) and for GM-CSF GM-MØ (dashed line). Elevated levels of CD14 antigen protein were detected in cells incubated with M-CSF while cells from either CSF culture stained positive for CD68 indicating phenotypic conversion into 2 subsets of macrophages designated as M-MØ (CD68+/CD14+) and GM-MØ (CD68+/CD14-), respectively. Similar results were obtained with another monocyte donor.

Supplementary Figure S3

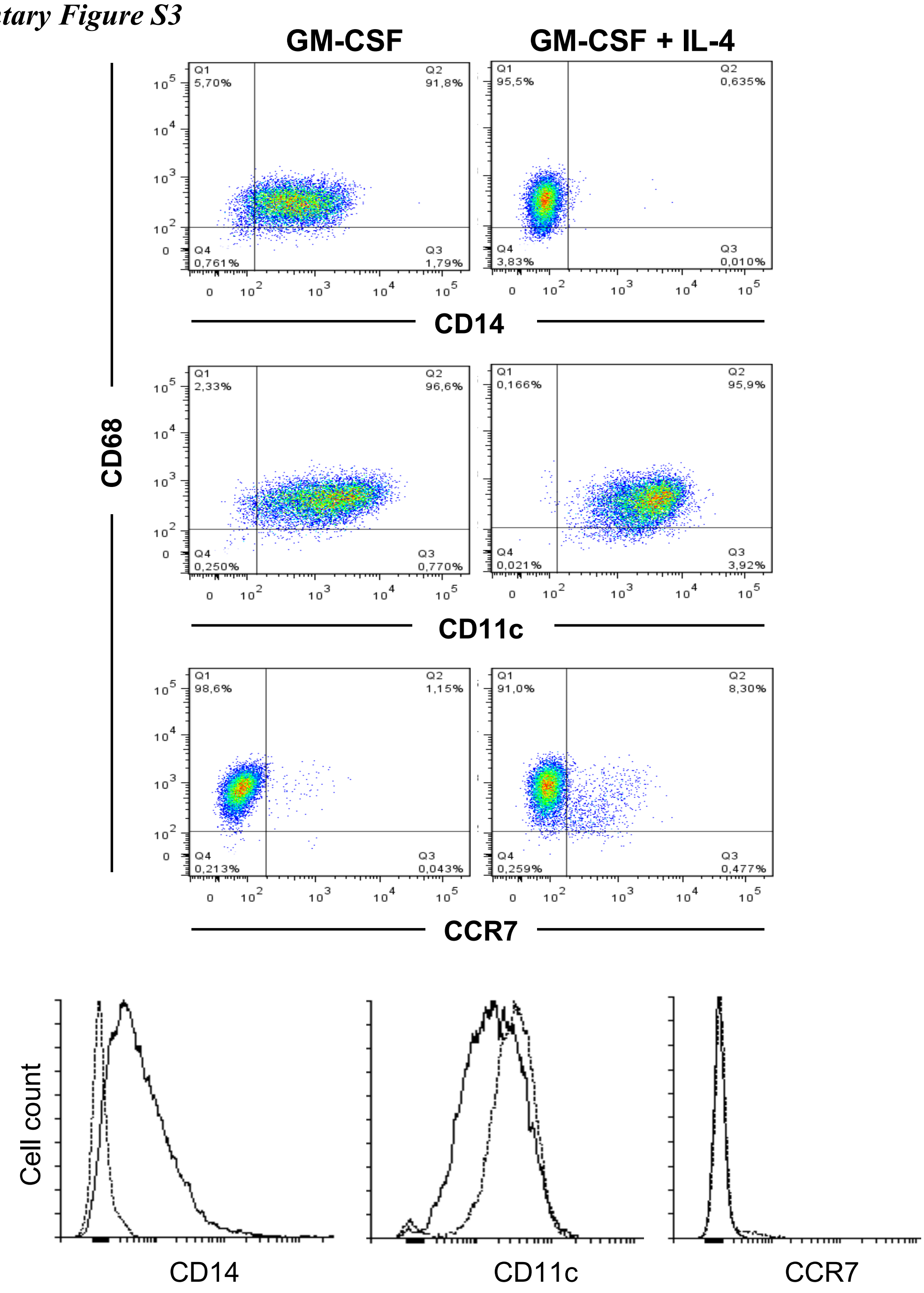

Figure legend S3. ***Expression of dendritic cell markers in monocytes differentiated in GM-CSF in the absence or presence of IL-4***

Cells were grown in the presence of GM-CSF or GM-CSF with IL-4, and analyzed for the expression of CD68 and CD14, and the common dendritic cell markers CD11c and CCR7 by flow cytometry to evaluate formation of any contaminating dendritic cells. **Top panel**: Quadrant plots were created to evaluate the proportion cells double-positive for CD68/CD14, CD68/CD11c and CD68/CCR7. Fluorescence thresholds for positive staining were set using isotype-matched immunoglobulins. **Bottom panel**: Overlaid histograms of antigen expression intensity in the CD68-positive cells generated in the presence of GM-MØ (solid line) or GM-CSF with IL-4 for dendritic cells (dashed line). The results indicate that GM-CSF-differentiated cells expressed the macrophage CD14 antigen. However, the presence of the cytokine IL-4 together with GM-CSF supported the generation of dendritic cells as seen by absence of CD14 and increased expression of CD11c, and a subpopulation of cells expressing the CCR7, typical of mature dendritic cells. Similar results were obtained with another monocyte donor.

Supplementary Figure S4

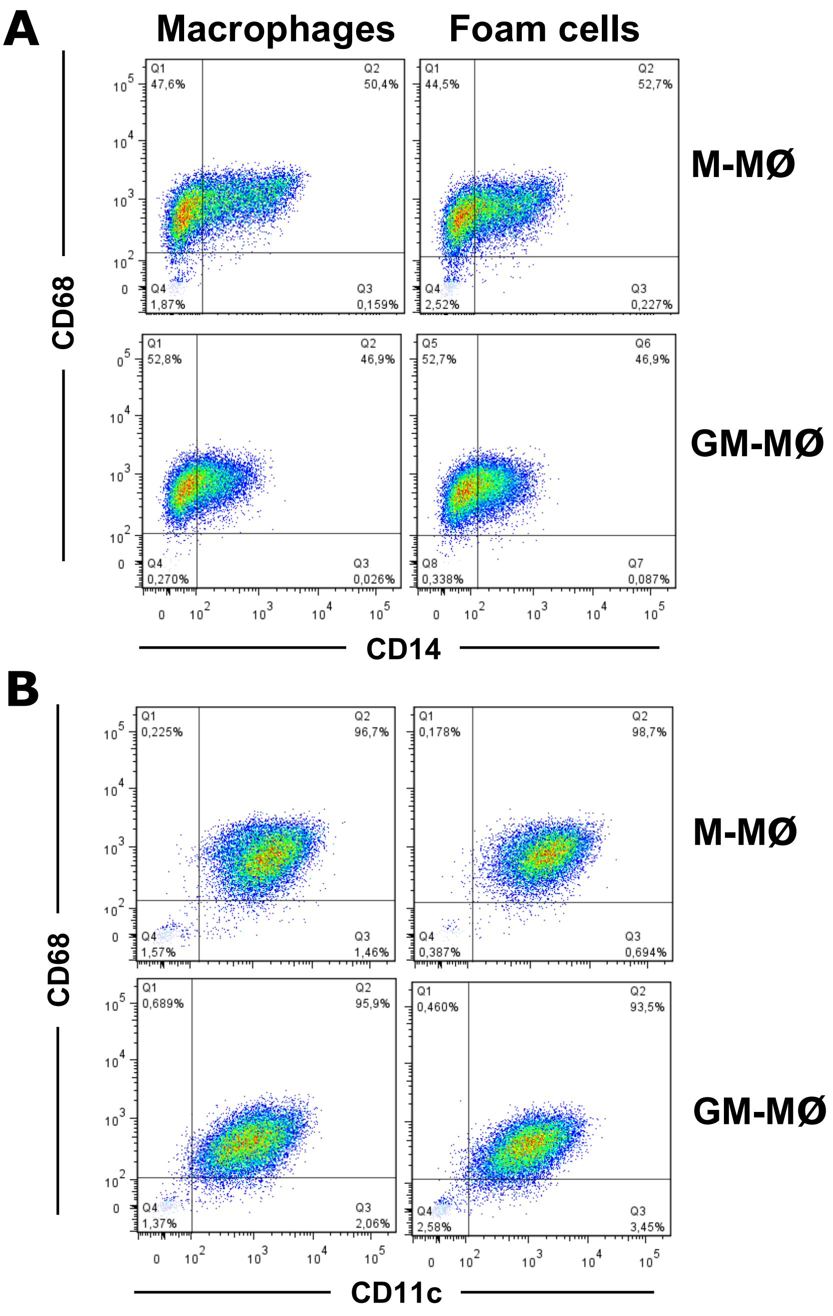

Figure legend S4. ***Expression of CD14 and CD68 proteins in M-MØ and GM- MØ foam cells***
Flow cytometry analysis of CD68, CD14, and CD11c in freshly differentiated M-MØ and GM-MØ (Macrophages) and after incubation of the cells for 24 h with 25 µg/ml of acetyl-LDL (Foam cells). Quadrant plots were created to evaluate the proportion cells double-positive for CD68/CD14 (***A***) and CD68/CD11c (***B***). Fluorescence thresholds for positive staining were set using isotype-matched immunoglobulins. The results indicated that cholesterol loading did not influence expression of CD14 and CD11c in either cell subtype. Data are representative of cells derived from 3 monocyte donors.

Supplementary Figure S5

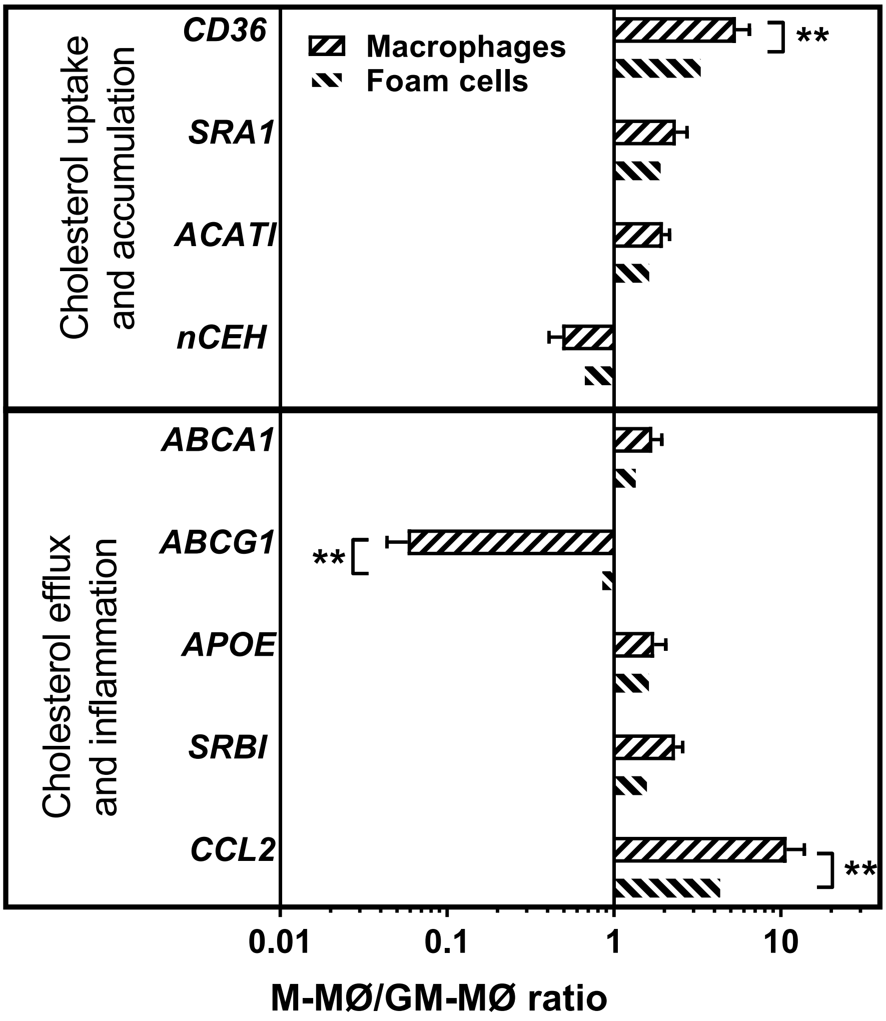

Figure legend S5. ***Effect of cholesterol loading on the differential expression of critical genes in non-foamy and foamy MØ of each CSF subtype***

The relative expression level of macrophage genes involved in cholesterol uptake and accumulation (***top panel***) and cholesterol efflux and inflammation (***lower panel***) was determined in non-loaded macrophages and macrophage foam cells by calculating the M-MØ/GM-MØ ratio for each gene shown in ***Figure 1*** and *SCARBI*, which encodes SR-BI receptor protein. Data (shown as means ± SEM) correspond to 10 monocyte donors of data shown in ***Figure 1***.

Supplementary Table SI. ***Characteristics of the LDL preparations***

LDL preparations	LDL-protein (mg/mL)	LDL-cholesterol (mg/mL)	Prot/Chol ratio	Particle size (nm)	TBARS (nM/mg protein)
n-LDL (A)*	8.5	15.5	0.55	28.5	0.42
n-LDL (B)*	9.0	16.9	0.53	29.1	0.18
ac-LDL	1.1	3.2	0.34	39.7	0.21
ox-LDL	1.0	1.6	0.63	27.6	41.2

*LDL batch

Supplementary Table S2. ***PCR primers and fluorogenic TaqMan probes used in the quantitative RT-PCR analysis (shown in 5´→ 3´orientation)***

**Gene**	**Forward primer**	**Reverse primer**	**Taq Man probe (if used)**
ABCA1	CATCTTTGGGACACCTCAGA	ATGAATTGTGCTGGGCATT	
ABCG1	CCATCCCCACGTACCTACA	GCAGATCTTCCCGGTCTAAG	
ACAT1	ATCATGATGGCCTCTCAAAGTC	GGACATGCTCTCCATCCCA	CCACCATCACATCCTGATGTCCACAC
ApoE	GAGCAGGCCCAGCAGATAC	CTTCGGCGTTCAGTGATTGT	
CD36	GGGAAAGTCACTGCGACATG	TGCAATACCTGGCTTTTCTCA	TTAATGGTACAGATGCAGCCTCATTTCCA
CXCL8	CTCTTGGCAGCCTTCCTGA	GGTGGAAAGGTTTGGAGTATGTC	TGGCAAAACTGCACCTTCACACAGAG
CCL2	CATAGCAGCCACCTTCATTCC	CTGCACTGAGATCTTCCTATTGG	CTCGCTCAGCCAGATGCAATCAATG
GAPDH	CCACATCGCTCAGACACCAT	GGGCAACAATATCCACTTTACCAGG	CCAATACGACCAAATCCGTTGACTCC
IL1B	TTACAGTGGCAATGAGGATGAC	GTCGGAGATTCGTAGCTGGAT	AACAGATGAAGTGCTCCTTCCAGGACC
LXRA	TGGGAGTGAGAGTATCACCTTCCT	CAAACTCGGCATCATTGAGTTG	TCCACTTGCAGCCCTGCTTTGG
nCEH	TCCTGGACTTGATAGCAGATGTG	GCTTGGACGGTACTGAAACTCA	CCACAGAGATGCTGGAGCACCCA
PPARA	GCTCGGTCATCACGGACAC	GTCCCCGCAGATTCTACATTC	AGCCCCTCCTCGGTGACTTATCCT
PPARG	GGTTTCAGAAATGCCTTGCA	GCTGGTCGATATCACTGGAGAT	CGCCCAAACCTGATGGCATTATGAG
SRA1	AGGATTTCCAGGTCCAATAGGTC	GAGTCCTCGACTTCCAGGAAAG	
TNFA	GCTGCACTTTGGAGTGATCG	GTTTGCTACAACATGGGCTACAG	CCCAGGCAGTCAGATCATCTTCTCGA
